# A structured approach to modifying an implementation package while scaling up a complex evidence‐based practice

**DOI:** 10.1111/1475-6773.14313

**Published:** 2024-05-15

**Authors:** Kristina M. Cordasco, Sonya E. Gabrielian, Jenny Barnard, Taylor Harris, Erin P. Finley

**Affiliations:** ^1^ VA Center for the Study of Healthcare Innovation, Implementation and Policy, VA Greater Los Angeles Healthcare System Los Angeles California USA; ^2^ Department of Medicine VA Greater Los Angeles Healthcare System Los Angeles California USA; ^3^ Department of Medicine David Geffen School of Medicine at the University of California at Los Angeles Los Angeles California USA; ^4^ Desert Pacific Mental Illness Research, Education, and Clinical Center (MIRECC), VA Greater Los Angeles Los Angeles California USA; ^5^ Department of Psychiatry VA Greater Los Angeles Healthcare System Los Angeles California USA; ^6^ Department of Psychiatry and Biobehavioral Sciences, David Geffen School of Medicine University of California at Los Angeles Los Angeles California USA; ^7^ National Center on Homelessness among Veterans (NCHAV) Los Angeles California USA; ^8^ Departments of Medicine and Psychiatry and Behavioral Sciences, Long School of Medicine University of Texas Health Science Center at San Antonio San Antonio Texas USA

**Keywords:** evidence‐based practice, health services administration, homelessness, implementation science, veterans

## Abstract

**Objective:**

To describe *a* structured, iterative, data‐driven approach for modifying implementation strategies for a complex evidence‐based practice during a nationwide scale‐up initiative.

**Data Sources and Study Setting:**

We scaled‐up implementation of Critical Time Intervention (CTI)—an evidence‐based case management model—across 32 diverse community‐based Veterans Affairs (VA) “Grant and Per Diem” case management (GPD‐CM) agencies that serve homeless‐experienced Veterans transitioning to independent living. Primary data were collected using qualitative methods.

**Study Design:**

We embarked on a scale‐up initiative while conducting a pragmatic randomized evaluation using a roll‐out design, comparing two versions of a CTI implementation package tailored to VA's GPD‐CM program. We iteratively assessed contextual factors and implementation outcomes (e.g., acceptability); findings informed package modifications that were characterized using the Framework for Reporting Adaptations and Modifications to Evidence‐based Implementation Strategies.

**Data Collection Methods:**

We conducted semi‐structured interviews with Veterans, GPD‐CM staff, and liaising VA clinicians; periodic reflections with liaising VA clinicians and implementation team members; and drew upon detailed meeting notes. We used rapid qualitative methods and content analysis to integrate data and characterize modifications.

**Principal Findings:**

After each scale‐up wave—in response to variations in agency‐level characteristics— we made iterative modifications to the implementation package to increase CTI adoption and fidelity across the diverse contexts of our scale‐up sites. Modifications included adding, deleting, integrating, and altering the package; core package components were preserved.

**Conclusions:**

Implementation packages for complex evidence‐based practices undergoing scale‐up in diverse contexts may benefit from iterative modifications to optimize practice adoption with fidelity. We offer a structured, pragmatic approach for iteratively identifying data‐driven, midstream implementation package adjustments, for use in both VA and non‐VA scale‐up initiatives. Our project demonstrates the importance of assessing for and making modifications in a scale‐up initiative, as well as the trade‐offs of projects having simultaneous formative and summative evaluation aims.

**What is known on this topic**

- Use of evidence‐based practices (EBPs) in healthcare and social services is suboptimal.
- Scaling‐up efforts to implement EBPs are challenged by variations in patient, clinician, organizational, and socioecological factors across settings.
- Making modifications to implementation strategies so that they align with the circumstances of the target sites is more likely to lead to successful outcomes.

**What this study adds**

- We provide an example of a pragmatic, structured approach to modifying an implementation package while rolling‐out a complex evidence‐based practice.
- We demonstrate why robust, iterative assessments, and as needed modifications of implementation strategies are essential for scale‐up initiatives, especially in the early phases.

## 1 INTRODUCTION

There is widescale agreement that a major impediment to improving the quality, equity, and outcomes of healthcare and social services are the challenges inherent to scaling up the use of evidence‐based practices (EBPs).^1^ When scaling up the implementation of an EBP, being responsive to contextual factors—influential features of the setting in which the EBP is being implemented—by modifying the implementation strategies so that they align with the circumstances of the target sites, increases the likelihood of successful outcomes.^2^ However, when embarking on a scale‐up initiative, implementers are often faced with having incomplete knowledge of the contextual factors for all sites that will be included in the effort. Contextual factors may also be dynamic or emergent over the course of a scale‐up initiative.^2^ Further, it is often not knowable in advance how these contextual factors will interact with the implementation strategies. In such cases, it is important that scale‐up initiatives use systematic, iterative, and efficient approaches for making midstream data‐driven modifications to implementation strategies in response to newly discovered or shifting contextual factors.^3^

With funding from VA's Quality Enhancement Research Initiative (QUERI), as a Partnered Implementation Initiative (PII), we sought to scale‐up the use of Critical Time Intervention (CTI) — an evidence‐based case management model for promoting housing retention— in Grant and Per Diem Case Management Program (GPD‐CM) agencies.^4^ GPD‐CM agencies are community‐based nonprofit organizations or state, local, or Indian Tribal government agencies; some are focused on delivering social services to Veterans while others serve multiple vulnerable populations. The VA GPD‐CM program provides 6 months of case management for homeless‐experienced Veterans transitioning to independent housing; the program is delivered by community‐based agencies with funding from VA. CTI is a good fit with the time‐limited, transitional nature of VA's GPD‐CM program in that, with CTI, case managers deliver field‐based services, structured into three time‐limited phases of decreasing service intensity, to enable homeless‐experienced Veterans to establish connections with VA and other community‐based resources that will provide them with long‐term supports for meeting their health and social needs.

Under QUERI's PII funding mechanism, the primary goal is implementation of EBPs, using and assessing the effectiveness of defined implementation strategies. With initial pilot PII funding, we developed and tested a CTI implementation package (i.e., resources and processes), consistent with the Replicating Effective Programs (REP) implementation framework,^5^ tailored to VA's GPD‐CM program. We then received QUERI funding to scale‐up CTI to an additional 32 GPD‐CM agencies nationally, with diverse contextual features, using a roll‐out design in sequential nine‐month waves.

In response to the multi‐level, dynamic contextual factors that emerged, via formative feedback, while scaling up implementation of CTI across GPD‐CM agencies, we iteratively modified our implementation package. We describe a structured, iterative, data‐driven approach for modifying an implementation package for a complex evidence‐based practice in the midst of a pragmatic scale‐up initiative employing a roll‐out design.

## 2 METHODS

### 2.1 Implementation approach and design

The development of our initial implementation package, with the pilot PII funding, is described elsewhere.^6^ Briefly, we built upon implementation approaches and materials used in other settings and engaged and garnered perspectives from diverse VA, GPD agency, and CTI experts and stakeholders. We then implemented and formatively evaluated materials and processes in four GPD‐CM agencies affiliated with a VA Medical Center (VAMC) in a major metropolitan area serving a large population of homeless‐experienced Veterans through a myriad of tailored VA and non‐VA medical and social services. Guided by REP and best practices in implementing psychosocial interventions, the resulting package—resources and processes for supporting the GPD‐CM staff in adopting and sustaining use of CTI—blended evidence‐based implementation strategies (which we refer to as “components”), consisting of initial orientation and training, community practice sessions,^7^ external facilitation,^8^ an online toolkit,^9^ case consultations,^10^ and an interactive email‐based listserv.^11^ These package components are described in Table 1. In the subsequent scale‐up phase, we tested and compared two versions of the implementation package, one augmented by external facilitation and one without, in a type 3 hybrid implementation‐effectiveness trial using a cluster‐randomized design, which has also been described elsewhere.^12^ We scaled‐up implementation of CTI to 32 additional agencies across three sequential scale‐up waves. For the first scale‐up wave, we utilized the implementation package as described in Table 1; for the two subsequent scale‐up waves, we used iteratively modified versions of the package. The implementation team for the scale‐up initiative consisted of community of practice leads (two social workers, trained in CTI, who presented content to augment and reinforce CTI knowledge and skills, organized speakers for sessions, and led group discussions), external facilitators, a toolkit coordinator, a project manager, and two principal investigators (PIs).

**TABLE 1:** Initial critical time intervention (CTI) implementation package.

Component	Description	Timing
Orientation	One 30‐min video conference introducing implementation team, training components, and their timing; printed CTI manual and written orientation materials mailed in advance	Implementation week 1
Intensive training	Six weekly 2‐h videoconference sessions of initial CTI training and train‐the‐trainer sessions for supervisors	Implementation weeks 2–7
Community of practice (CoP) sessions	Twice monthly, 1‐h videoconference sessions for reinforcing CTI training, continuing to build case managers' CTI knowledge and skills, and discussing challenges that are encountered.	Implementation weeks 8–40
External facilitation sessions	Weekly 30‐min videoconference sessions, facilitated by a member of our implementation team, for agency case managers and supervisors to discuss and troubleshoot agency‐specific implementation challenges	Implementation weeks 8–40 (Only agencies assigned to receive this component)
Online CTI toolkit	Online resources and tools including forms to support CTI implementation, the CTI training manual, and recordings of training and CoP sessions	Implementation weeks 1–40
Case consultations	On‐demand 30‐min telephone or videoconference consultations with a CTI expert to discuss challenges encountered using CTI with individual clients	Implementation weeks 8–40
Email‐based listserv	Case managers and supervisors asynchronously asking questions about and sharing their CTI clinical and implementation practices	Implementation weeks 8–40

### 2.2 Evaluation approach and design

The stakeholder engagement and formative evaluation design for the pilot, as well as our implementation evaluation methods for the scale‐up, have also been described elsewhere.^6^, ^12^ Briefly, to iteratively evaluate our implementation package through the scale‐up phase, a qualitative evaluation team, separate from the implementation team, conducted semi‐structured interviews with GPD‐CM case managers and supervisors and VA staff serving as liaisons to the GPD‐CM agencies (referred to as “GPD‐CM liaisons”) to assess common contextual factors (guided by the Consolidated Framework for Implementation Research)^13^ and implementation outcomes (guided by the Proctor taxonomy of implementation outcomes).^14^ The evaluation team also conducted periodic reflections, which are brief guided discussions intended to capture dynamic implementation activities, events, and context over time, with GPD‐CM liaisons (quarterly) and implementation team members (monthly; conducted separately with the PIs and project manager, external facilitators, and community of practice leads).^15^ Reflections were documented using near‐verbatim notes and/or recorded and transcribed using Microsoft Teams. Separately, the evaluation team documented implementation challenges described during Community of Practice sessions with detailed notes. Rapid qualitative analysis methods were used to summarize findings associated with Veteran and GPD‐CM staff experiences and perspectives,^16^ including perceptions of CTI and implementation package components (e.g., training, external facilitation); organizational (e.g., supervisor engagement) and socioecological features (e.g., rural/urban) at each agency; and interactions with broader societal shifts (e.g., employment patterns as the United States emerged from the Coronavirus Disease 2019 [COVID‐19] worldwide pandemic).^17^ The qualitative evaluation team, consisting of an administrator and five master's‐ or PhD‐trained qualitative methodologists and led by our third PI, met weekly via videoconference to coordinate activities as well as discuss and synthesize the information as they were obtaining it, targeting topics that would be most useful for the implementation team to receive promptly. This project was determined to be non‐research by VA's Central Institutional Review Board.

### 2.3 Iterative modification approach and design

**FIGURE 1:**
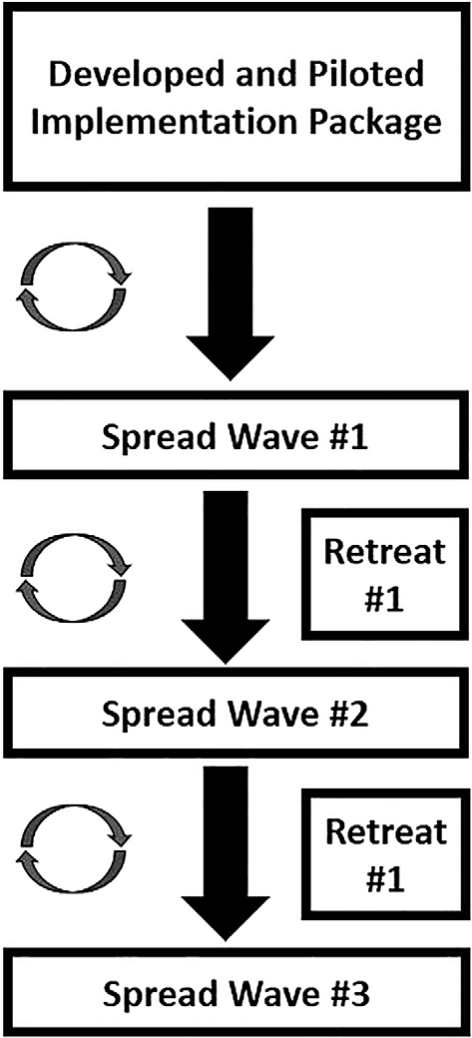
Implementation waves and retreats.

Implementation package modifications were selected and formulated via implementation leaders and practitioners, in collaboration with evaluators, and occurred both during and between scale‐up waves. Modifications were informed by information emerging from two primary sources: implementation team interactions with agencies, and evaluation team activities. The figure shows the design we used for making iterative modifications, as detailed below (Figure 1).

Modifications were made throughout the scale‐up waves. The implementation team met via videoconference weekly to report on implementation progress and challenges and make decisions about potential modifications. To support these decisions, salient information was exchanged near real‐time between the implementation and evaluation teams during weekly videoconference meetings of the PIs, which also included the overall project director. As needed, the implementation team reached out to the evaluation team for time‐sensitive information related to specific topics (e.g., the online toolkit).

More substantial implementation package modifications were made between scale‐up waves. To consider these modifications, we held two one‐day in‐person retreats combining the implementation and evaluation teams; the first retreat was held between scale‐up waves 1 and 2, and the second one was held between scale‐up waves 2 and 3. During these retreats, sequential discussions focused on each implementation package component. For each component, the implementation team briefly described current resources and processes for the component, including the challenges they were encountering. Next, the qualitative team presented related evaluation findings, including participant feedback on acceptability and usability, and any information they had relevant to the implementation challenges being encountered. The implementation and evaluation teams then collaboratively discussed the current state of the component and possible revisions and reorganizations. After each retreat, based on these discussions, the implementation team formed time‐limited task‐focused workgroups to make modifications to the materials and processes.

### 2.4 Documenting modifications

During the scale‐up waves, modifications made to the implementation package were documented via periodic reflections conducted with the implementation team members (PIs, external facilitators, and community of practice leads);^15^ modifications made between waves were captured in retreat notes. To compile these modifications, and their contexts and rationales, the evaluation team conducted a content analysis of these reflections and notes, resulting in a preliminary set of modifications with relevant information related to context and/or implementation outcomes (e.g., acceptability); the lead author then merged and synthesized the list of modifications from across data sources. Using the Framework for Reporting Adaptations and Modifications to Evidence‐based Implementation Strategies (FRAME‐IS),^18^ for each modification, the lead author documented which component was modified, how and when it was modified, and the rationale and goal, followed by member‐checking with the implementation team to confirm that modifications were accurately captured.^19^

## 3 RESULTS

Across scale‐up waves, we implemented CTI in 32 GPD‐CM agencies, with varying organizational and community contexts, spanning 18 states and three geographic regions of the United States (Table 2). These agencies were affiliated with 28 VAMCs, which varied in the services they had available for homeless‐experienced Veterans; for example, approximately half had VA Homeless Patient Aligned Care Teams, which are primary care teams tailored and enhanced to meet the needs of homeless‐experienced Veterans.^20^

**TABLE 2:** Characteristics of the grant and per diem case management (GPD‐CM) agencies and their associated VA medical centers (VAMCs), by wave.

Agency characteristics					
	Pilot Wave (*n* = 4)	Spread Wave 1 (*n* = 11)	Spread Wave 2 (*n* = 11)	Spread Wave 3 (*n* = 10)	All Waves (*n* = 36)
Geographic region^a^					
Northeast	0 (0%)	3 (27%)	3 (27%)	1 (10%)	7 (19%)
Midwest	0 (0%)	3 (27%)	2 (18%)	5 (50%)	10 (28%)
West	4 (100%)	5 (45%)	6 (55%)	4 (40%)	19 (53%)
Rural community^b^	0 (0%)	2 (25%)	2 (25%)	0 (0%)	4 (16%)
Type					
Non‐profit	4 (100%)	10 (91%)	10 (91%)	9 (90%)	33 (92%)
Government	0 (0%)	1 (9%)	1 (9%)	1 (10%)	3 (8%)
Population focus					
Veterans only	2 (50%)	6 (55%)	4 (36%)	5 (50%)	17 (47%)
Veterans and non‐Veterans	2 (50%)	5 (45%)	7 (64%)	5 (50%)	19 (53%)
GPD‐CM case managers per agency^c^					
<1	0 (0%)	0 (0%)	0 (0%)	1 (10%)	1 (3%)
1	1 (25%)	8 (73%)	8 (73%)	7 (70%)^d^	24 (67%)
2	1 (25%)	2 (18%)	1 (9%)	2 (20%)	6 (17%)
3	2 (50%)	1 (9%)	2 (18%)	0 (0%)	5 (14%)
Had turnover/vacancies in one or more positions^e^	2 (50%)^f^	7 (64%)^g^	6 (55%)^h^	2 (22%)^i^	15 (42%)

Associated VAMC characteristics					
	Pilot Wave (*n* = 1)	Spread Wave 1 (*n* = 8)	Spread Wave 2 (*n* = 10)	Spread Wave 3 (*n* = 9)	All Waves (*n* = 28)^j^
Have HPACTs^k^	1 (100%)	5 (63%)	6 (60%)	6 (67%)	16 (57%)

VAMC complexity levels^l^					
1a	1 (100%)	4 (50%)	4 (40%)	3 (22%)	12 (43%)
1b	0 (0%)	1 (13%)	2 (20%)	2 (33%)	5 (18%)
1c	0 (0%)	1 (13%)	0 (0%)	1 (11%)	2 (7%)
2	0 (0%)	0 (0%)	1 (10%)	0 (0%)	1 (4%)
3	0 (0%)	2 (25%)	3 (30%)	3 (33%)	8 (29%)

^a^ United States Census Regions (www.census.gov).

^b^ Per the United States Department of Agriculture Rural‐Urban Commuting Area Codes (www.ers.usda.gov).

^c^ Fulltime equivalents (FTEs) funded by Grant and Per Diem Case Management (GPD‐CM) program.

^d^ Includes one agency with 0.8 FTE funded.

^e^ Case manager or supervisor position changed staff members, or the position became vacant.

^f^ Two agencies had 1 position turnover.

^g^ One agency had 4 positions turnover, one had 3 positions turnover, two had 2 positions turnover, three had 1 position turnover.

^h^ One agency had 3 positions turnover, two had 2 positions turnover, three had 1 position turnover.

^i^ Two agencies had 2 positions turnover in the first half of implementation wave.

^j^ Some VAMCs repeated across waves.

^k^ Homeless Patient Aligned Care Teams: Primary care services with enhanced ancillary services tailored to meet the needs of homeless‐experienced Veterans^21.^

^l^ VA classifies its VAMCs as being high, medium, or low complexity based on patient population, clinical services availability, and education and research activities.

### 3.1 Scale‐up wave one

For our first scale‐up phase, we implemented CTI in 11 GPD‐CM agencies. As shown in Table 3, during this wave, we made multiple modifications to the implementation package, across components: intensive training; community of practice sessions; external facilitation; the online CTI toolkit; and broader modifications that impacted all components. The most significant modifications were motivated by our experience with high turnover among case managers and supervisors; a lack of clinical training among supervisors; large variations in case manager clinical training and experience; and case managers and supervisors generally possessing low levels of knowledge about VA services and processes. In response, we developed and implemented monthly orientations, added coaching about and resources for foundational case management skills, and developed a “Resources and Processes Workbook” to guide GPD‐CM staff in collecting and documenting information about their local VA and community resources. We also collected data and received feedback that our online toolkit was being utilized and was highly valued; however, some individuals expressed not knowing where to find some of the forms. Therefore, we further developed resources for the toolkit and refined its navigation. Finally, we found that case managers used different terms when referring to the GPD‐CM program and therefore needed to adjust our package to improve the clarity of our communications. Most commonly, modifications added elements to the package components (e.g., adding sessions and resources), but we also integrated elements, deleted one component, and refined the existing package. The goals for modifications were to increase the acceptability, appropriateness, and feasibility of the implementation package as well as to increase fidelity to and clinical effectiveness of CTI.

**TABLE 3:** Salient modifications to the implementation package components.

Component modified	Description of modification(s)	Modification type	Reason(s)
**Wave 1**			
Orientation	Developed materials for and launched monthly orientation sessions	Adding	There was frequent turnover among GPD‐CM^a^ staff (case managers and supervisors); Staff members starting mid‐wave needed to be oriented to CTI^b^ and the program.
Intensive training	Decided not to provide supervisors with CTI “train‐the‐trainer” training to deliver this component in the sustainment phase	Deleting	There was frequent turnover in GPD‐CM supervisors, and some supervisors were not clinical.
Community of practice sessions	Provided links to relevant forms and resources in online toolkit during community of practice sessions	Integrating	GPD‐CM staff were indicating that they did not know about the availability, or how to find, these forms and resources.
Community of practice sessions	Increased information provided about available VA services	Adding	GPD‐CM staff had low levels of knowledge about services VA provides.
External facilitation	Added coaching about (and resources for) case manager knowledge/skills that are foundational to using CTI	Adding	GPD‐CM staff were highly variable in their training and experience; some supervisors were nonclinical.
Toolkit	Further developed and refined online toolkit to include more information and resources; improved layout and provided guidance on navigating toolkit resources	Adding	GPD‐CM staff found forms and other written tools very useful and were asking for these resources; created durable materials to be used in the sustainment phase.
Toolkit	Developed “Resources and Processes Workbook” to guide GPD‐CM staff in collecting and documenting information about their local VA and community resources	Adding	GPD‐CM staff did not know the details of, nor how to navigate, the services available at their affiliated VAs and in their communities; local program knowledge was often lost with turnover in staff.
All components	Changed terminology to refer to program as “GPD Aftercare Program” across all components	Packaging	GPD‐CM staff at some agencies were confused by the use of the formal name “GPD Case Management Program” as this is not how they referred to it.
**Wave 2**			
Community of practice sessions	Incorporated case presentations; provided content reinforcing intensive training; built in more time for questions and interactive discussion about CTI skills; heightened focus on importance of and procedures for supervision	Integrating	In response to retreat #1 discussion: Wave 1 GPD‐CM staff were highly variable in their training and experience; some supervisors were nonclinical; case managers were not using on‐demand case consultations or listserv.
Case consultation	Added scheduled drop‐in hours when case managers could bring cases for troubleshooting assistance and/or learn from others' cases others	Adding	In response to retreat #1 discussion: On‐demand case consultations were being suboptimally used.
Toolkit	Continued expanding and refining toolkit resources	Adding	In response to retreat #1 discussion: With high turnover in GPD‐CM staff, online toolkit resources were essential for orienting and educating new staff.
Listserv	Stopped encouraging use of listserv for GPD staff to asynchronously ask questions or engage in discussion	Deleting	In response to retreat #1 discussion: GPD staff at wave 1 agencies had not been using listserv to ask questions of engage in discussion (incorporated time for questions and interactive discussion into Community of Practice Sessions).
External facilitation	Added emphasis on the importance and role of supervisors in CTI and assisted agencies in identifying a clinical supervisor	Adding	Case managers were getting suboptimal supervisors support and some supervisors were nonclinical.
**Wave 3**			
Orientation	Provided further foundational information about CTI and the trainings/resources available	Refining	In response to retreat #2 discussion: Some GPD staff members expressed confusion about CTI and trainings/resources available.
Toolkit	Created implementation roadmaps, with directed discrete activities to guide case managers and supervisors in implementing CTI	Refining	In response to retreat #2 discussion: Some wave 2 GPD staff expressed uncertainty about how to get started with implementing CTI. GPD staff were very focused on forms, found the forms and other written tools very useful, and were asking for more resources.
All components	Changed terminology to again refer to program as “GPD Aftercare Program,” and clarified that some used term “aftercare” or “housing retention program”	Packaging	GPD staff at some agencies were confused by the use of the term “Aftercare Program”, as they used the formal name, or “housing retention program” instead.

^a^ Grant and Per Diem Case Management Program.

^b^ Critical Time Intervention.

### 3.2 Scale‐up wave two

With scale‐up wave two, we implemented CTI in an additional 11 GPD‐CM agencies. At the onset of this wave, we made three significant modifications, based on formative data from wave one and our first retreat (Table 3). The motivating factors for these modifications were the same as those made in the midst of wave one. In addition, two of our implementation package components—on‐demand case consultations and the listserv—had low utilization. Therefore, we deleted the listserv component and, in its place, integrated case discussions into our community of practice sessions and allowed time for case managers to ask questions and share information. We created a new implementation component, “drop‐in hours”—an optional biweekly videoconference session—for GPD‐CM case managers and supervisors to discuss cases and answer questions, as needed. We also continued expanding and refining toolkit resources. The main goal of these modifications was to increase fidelity to CTI.

### 3.3 Scale‐up wave three

Based on the discussions from our second retreat, in scale‐up wave three we built upon and further refined the modifications made in the previous waves while implementing in our final 10 GPD‐CM agencies (Table 3). Implementation package modifications for this wave included further refining the orientation as well as the online toolkit to further increase CTI fidelity and sustain it in the context of ongoing GPD‐CM staff turnover. We also needed to further refine the terminology we were using to refer to the GPD‐CM program as we discovered that the GPD‐CM staff in this wave used the original, or different, terms to refer to the program.

## 4 DISCUSSION

In our scale‐up initiative of CTI in VA's GPD‐CM program, we iteratively modified our implementation package in response to emerging information on relevant characteristics and conditions for our scale‐up agencies, as well as how agencies were interacting with our implementation package. In addition, the situation was dynamic in that, as society emerged from the COVID‐19 pandemic, there were record low levels of unemployment nationally resulting in higher turnover in staff across most employment sectors. These circumstances resulted in our needing to make modifications to our implementation components concurrent to using them; we responded by developing a structured, iterative, data‐driven, and efficient approach for making midstream adjustments to our implementation package.

The “Framework for Going to Full Scale” describes a four‐phased approach for scaling‐up initiatives.^3^ With this approach, in the first two phases, implementers set up and then develop and test the EBP implementation package within a single demonstration site, as was done in our pilot. The third phase is the “test of scale‐up,” in which the package is used to scale‐up the EBP beyond its first demonstration site to a cohort of other sites with contexts that are representative of the population of potential sites. Our scale‐up to 32 GPD‐CM agencies—using a roll‐out design with 10–11 agencies per wave—was in line with this “test of scale‐up” phase, providing an example of efficiently and iteratively generating information and responding with modifications during this testing phase, so as to promote and optimize the conditions for moving to the final phase, which is full‐scale implementation.

Our implementation initiative, the modifications we made to our implementation package, and the reasons for those adjustments, reinforce the value of having a scale‐up test phase^3^ as well as using iterations in this phase. Although the implementation package was developed and initially tested across four agencies, these agencies were associated with a single VA medical center with homeless and community resources that were not representative of most settings for the GPD‐CM program. Salient contextual factors, and how they would interact with our implementation package, became apparent only once we expanded beyond our initial pilot to scale‐up wave one agencies. With subsequent waves of heterogenic agencies, we were able to assess how our modified implementation package worked, rapidly integrate findings related to implementation challenges and outcomes, and make additional refinements. As might be expected, the number and scope of modifications decreased with each iteration; while we were adding and integrating components and elements in the first two waves, by the final wave, we were only making refinements to these components and elements.

Implementers must remain mindful that, during the test of scale‐up, they are preparing for a full scale‐up phase.^3^ Therefore, for each potential modification, the implementation team considered whether processes would be feasible for the full scale‐up phase. In addition, to prepare for full scale‐up, we worked in close partnership with VA national homeless services leaders to create implementation processes that aligned with overall homeless program efforts and priorities.

Modifications were aimed at optimizing both initial implementation effectiveness and sustainment. As described in the Integrated Sustainability Framework, EBP sustainment is influenced not only by the characteristics of the EBP and implementers, but also by the processes used to implement, as well as organizational and environmental contextual factors.^21^ Engaging in a test of scale‐up across contextually heterogenic sites, and making responsive modifications to implementation processes, may increase the likelihood of EBPs being sustained.^2^

A major factor driving our modifications was the high turnover of the case managers and supervisors in the GPD‐CM agencies, which is an example of a dynamic contextual factor. This circumstance illustrates how, when doing the test of scale‐up as well as going to full‐scale, it is important to set up systems for ongoing assessments to ensure that the implementation package remains aligned with potentially evolving contextual factors; for example, by using pragmatic and rapid methods, such as periodic reflections and/or check‐ins.^15^ This monitoring for and responding to shifts in contextual factors, which, in other cases, may be more subtle than the employment turnover we observed within this initiative, is also paramount for successful maintenance of implementation after scale‐up.

Foundational to our approach was the implementation team having reliable and timely information from our evaluation team to guide package modifications. Reliable data‐driven information allows teams to make informed decisions about potential modifications to their implementation strategies^22^; information being timely enables implementers to address challenges as they arise. To ensure that the information was reliable, our evaluation team used rigorous qualitative methods, gathering data from a multitude of stakeholders—case managers, supervisors, GPD liaisons, and Veterans. To provide timely information, they used rapid qualitative methods to efficiently identify and distill major themes and trends,^16^ and then synthesized this information and communicated it to the implementation team to inform modifications.^23^ It is important to note that the evaluation team was sufficiently robust, and distinct from the implementation team, demonstrating how scale‐up initiatives need to both plan for and be resourced for collecting, synthesizing, and then acting on information to modify their implementation strategies. There also needs to be intentional building and maintaining of collaborative relationships, with effective and efficient mechanisms for communication, between the implementing and evaluating teams, as we did with weekly scheduled meetings between the implementation and evaluation PIs, combined with retreats involving the entirety of both teams.

For scale‐up initiatives to have robust implementation and evaluation teams, as well as time to do iterative testing and modifications, there needs to be intentional investment of adequate resources for this purpose. However, traditional research grant funding mechanisms, scientific review panels, and IRB requirements are generally not well suited for formative assessments with midstream iterative modifications.^24^ QUERI, a VA funding entity whose mission includes “implementing evidence‐based practices and promising innovations into routine care,” is optimally aligned for supporting scale‐up initiatives in VA.^25^ QUERI, and its partnered implementation initiative mechanism, can thus serve as a model for other entities endeavoring to foster scale‐up of EBPs.

It is important to note that, while serving as a test of scale‐up, we were also engaging in a type 3 implementation‐effectiveness randomized evaluation of two versions of our implementation package—with and without external facilitation. Therefore, while making modifications, we were mindful to maintain the core elements of the two versions of the implementation package, as well as to keep them distinct (i.e., not to introduce elements of external facilitation into the other components). While it may be efficient to conduct a summative randomized evaluation of implementation strategies concurrent with a formative test‐of‐scale‐up, a drawback is that it constrains the implementation team's efforts to optimize the implementation strategies. Further, including in the summative evaluation agencies that received the implementation package prior to the modifications produces a bias to the null hypothesis (i.e., that there is no difference between the two versions of the implementation package) and increases the probability of making a type 2 error (i.e., finding no difference where one exists). Therefore, a concurrent formative and summative evaluation design may have detrimental effects on both the science as well as decisions related to preparing for the full‐scale phase. A better approach may be to focus on rigorous iterative formative evaluation for the initial scale‐up waves, and then follow this with a summative evaluation.

While our approach to modifying our implementation package has its strengths, its limitations include it only being applicable to scale‐up initiatives with adequate funding for robust implementation and evaluation teams. Further, we do not have empirical data that the approach we took to modifications was more effective or efficient compared with alternative approaches, although it was a good fit for the needs of our challenging scale‐up effort.

In summary, we provide an example of a pragmatic, structured approach to modifying an implementation package while rolling‐out a complex evidence‐based practice. This approach is appropriate for use in future scale‐up initiatives, within and outside VA. Moreover, our project demonstrates the paramount role of iterative testing and modification of implementation strategies, so as to optimize implementation strategies, when embarking on scale‐up initiatives.
